# Effects of Glycyrrhiza Polysaccharides on Chickens' Intestinal Health and Homeostasis

**DOI:** 10.3389/fvets.2022.891429

**Published:** 2022-05-12

**Authors:** Yu Wu, Chenyang Wu, Yanyun Che, Tao Zhang, Chen Dai, Audrey D. Nguyễn, Kun Duan, Yanyu Huang, Nannan Li, Hui Zhou, Xin Wan, Yuedi Wang, Hongjun Lei, Ping Hao, Caiyue Li, Yi Wu

**Affiliations:** ^1^Institute of Traditional Chinese Veterinary Medicine, College of Veterinary Medicine, Nanjing Agricultural University, Nanjing, China; ^2^Beijing Key Laboratory of Traditional Chinese Veterinary Medicine, Beijing University of Agriculture, Beijing, China; ^3^Engineering Laboratory for National Healthcare Theories and Products of Yunnan Province, College of Pharmaceutical Science, Yunnan University of Chinese Medicine, Kunming, China; ^4^College of Animal Science and Technology, Nanjing Agricultural University, Nanjing, China; ^5^College of Life Sciences, Experimental Teaching Center of Life Science, Nanjing Agricultural University, Nanjing, China; ^6^Department of Biochemistry and Molecular Medicine, School of Medicine, University of California, Davis, Sacramento, CA, United States; ^7^China Tobacco Henan Industrial Co., Ltd., Zhengzhou, China

**Keywords:** glycyrrhiza polysaccharides, gut immunity, intestinal barrier, gut microbes, feed additives

## Abstract

The overuse of antibiotics in poultry farming causes the accumulation of drug residue in animals' bodies and the occurrence of antibiotic-resistant bacteria, which not only compromise animals' health but ultimately endanger human health. Thus, there is an urgent need for a novel poultry feed additive to substitute for excessive antibiotics. Glycyrrhiza polysaccharides (GPS) derived from Chinese licorice have shown promising immunomodulatory effects in previous studies. The present study investigated the pharmacological effects of GPS on poultry intestines to assess whether it can be used as a feed additive. The results show that GPS can increase production of sIgA, promote the secretion activity of goblet cells, alter the gut microbial composition and lead to changes in short-chain fatty acids. GPS also elevated both Th1 and Th2 immune responses by facilitating the expression of IL-2, IL-4, IL-1β, and IFN-γ while increasing the proportion of both CD4+ and CD8+ cells in the intestine. Moreover, the results of 16S rRNA gene sequencing showed that GPS could significantly change intestinal microbiota composition in the intestine, evidenced by the increased proportion of Bacteroides, Butyricicoccus and Eisenbergiella, as well as a decreased portion of Erysipelatoclostridium, leading to a healthier intestinal microbiota composition for the host. Taken together, it can be concluded that GPS is safe to use as a novel feed additive that can be used as an alternative to prophylactic antibiotics in poultry feeding.

## Introduction

In recent decades, the global poultry farming industry developed rapidly in response to accelerating consumer demand. There are numerous feed additives used in industrial livestock and poultry husbandry to improve productivity, maintain intestinal health, and prevent pathogenic microorganisms (1, 2). Low-cost antibiotics are widely used in industrial farming to prevent bacterial infection and promote animal survival. However, abuse of antibiotics causes a number of unintended consequences, including drug residue in animal products and antibiotic-resistant microbes, both of which can damage human health (3). New research indicates the global death toll from such antimicrobial resistance in 2019 was upward of four million people 47. Therefore, there is an urgent need to find a novel feed additive to substitute for the overuse of antibiotics in industrial poultry farming.

The gastrointestinal tract is the first line of defense against the many considerable challenges posed by harmful pathogens ingested daily. If the health and balance of the intestine is disrupted, the entire body can become infected with pathogens. Therefore, supporting intestinal health is key to maintaining overall animal health. It is well-documented that the intestine has an expansive surface area in which microbiota thrive and interact with the immune system to achieve intestinal homeostasis and maintain host health (4, 5). SIgA, the dominant antibody found in intestinal mucosa, protects the intestine from pathogens and regulates the intestinal microbiota during its development from birth to adulthood (6, 7). In animals, sIgA deficiency can lead to immunodeficiency and incomplete intestinal barrier function, resulting in reduced poultry productivity and even death (7). Based on this, improving the level of sIgA in animals' intestinal mucosa is a useful strategy to maintain the intestine's homeostasis and host health (8).

Licorice, a traditional medicinal and food plant (9), has a long history of application in both diet and pharmacology (10). The edible and medicinal parts of licorice are the root and rhizome. Because of its sweet taste, its soaking liquid is often used as a drinking additive in China, and as a food sweetener in Europe (11). In recent years, more and more active ingredients in licorice such as polysaccharides, flavones, and saponins have been obtained, showing some potential effects in drug development and food industry (12). Glycyrrhiza polysaccharides (GPS) in particular show great potential for use in medical and food industries. GPS consist of a series of biological polysaccharides, mainly glucose, galactose, and mannose (13). Many studies have confirmed that GPS possess various pharmaceutical properties, acting as an antitumor (14), antibacterial (15), antioxidant (16), and immunomodulation (14) agent. It has also been reported that GPS could be used as an immunopotentiator, as it can directly facilitate a robust immune response to inoculation by promoting the proliferation of lymphocytes, especially T lymphocytes (13). Additionally, GPS can activate innate cells, such as macrophages and dendritic cells (DCs), which prime an immune response that can efficiently eliminate pathogens and senescent cells (17, 18). However, the effectiveness of GPS in maintaining intestinal health and regulating intestinal microbiota is still unknown.

Previous studies did not examine the question of GPS as a promoter of intestinal health, nor its potential ability as an immunopotentiator specifically for an intestinal immune response. The present study investigated whether feeding poultry GPS could benefit their overall intestinal health and beneficially regulate their intestinal microbiota. GPS was extracted and purified from Chinese licorice, then fed to male Hy-Line Brown chickens. Next, the effect of GPS on the intestine was explored and its mechanisms investigated.

## Materials and Methods

### Animals

Male 1-day-old Hy-Line Brown roosters were bought from Hai'an Shuangli Hatchery (Jiangsu, China). All animals were housed in wire cages under 12-h light/dark cycles at 30°C. All animal experiments were conducted in accordance with the guidelines of the Nanjing Agricultural University Institutional Animal Care and Use Committee (IACUC), detailed in the IACUC-approved protocol (No.:2021BAD34B04).

After one week of acclimatization, the roosters were randomly divided into four groups (*n* = 5) and treated either with isotonic saline (control group), GPS at a high dose (GPS-H, 600 mg/kg), GPS at a medium dose (GPS-M, 450 mg/kg), or GPS at a low dose (GPS-L, 300 mg/kg). The roosters in all groups were given these formulations intragastrically for 14 days. Meanwhile, the body weight of all roosters was measured every day to determine the safety of GPS. On the 15th day, all roosters were sacrificed and their contents, small intestine, bursa of Fabricius, spleen, and thymus were collected for further examination. The contents were directly transferred to −80°C for cryopreservation. The small intestine, bursa of Fabricius, bursa, and spleen were fixed with tissue fixative and stored under normal temperature conditions.

### Measurement of SIgA in the Intestine

On day 15, 8 cm of the duodenum from each rooster (*n* = 5 per group) was collected and washed eight times with PBS (phosphate buffer solution, pH = 7.4) containing phenylmethylsulfonyl fluoride (PMSF) protease inhibitor. Next, these solutions were centrifuged at 10,000 rpm for 15 min at 4°C. Afterward, the supernatant was collected and used to determine the level of sIgA in the intestines using an enzyme-linked immunosorbent assay (ELISA).

### Determination of Cytokines in Jejunum

The jejunums of 5 roosters from each group were collected, homogenized, then centrifuged at 12,000 rpm for 10 min at 4°C. Afterward, the supernatant was collected to analyze IL-2, IL-4, IL-1β, and IFN-γ levels in the intestine using ELISA kits.

### Immunofluorescence of Jejunum

On day 15, all jejunums were collected to detect proportions of CD4+ and CD8+ T cells. Jejunums were briefly incubated in primary antibodies CD4+ and CD8+. Then, secondary antibodies CY3 (red) and FITC (green) were incubated with the mixture for 1 h to stain CD4+ and CD8+, respectively. After rinsing, the expression of these markers in the specimen images was observed and captured using confocal microscopy.

### Histopathological Analysis

The paraffin-embedded bursa of Fabricius, spleen, thymus, liver, kidney, duodenum, jejunum, and ileum were sectioned using a pathology slicer (Leica RM2016), and a hematoxylin and eosin (HE) stain was applied to the sectioned tissue. Then, the stained intestinal segments were observed by using a Nikon Eclipse 80i microscope.

### Determination of Short-Chain Fatty Acids (SCFAs) in the Intestine

The contents samples from the intestine were collected to measure the level of short-chain fatty acids (SCFAs) using gas chromatography (GC) and then calculate standard curves. The contents of the cecum were vortexed with ultrapure water at a 1:5 ratio. Then, the mixture was centrifuged at 10,000 rpm for 10 min at 4°C and the supernatant was collected. Afterward, each supernatant was mixed 1:1 with 2-methyl butyric acid. The treatment of each sample and the GC analysis were conducted based on the product instructions (19).

### ABPAS Stain Assay

The jejunums were embedded in paraffin and sectioned using a Leica RM2016 pathology slicer. Then, the sections were sequentially treated with Periodic Acid Solution, Schiff reagent, and hematoxylin. After rinsing, the stained sections of the jejunums were observed by using a Nikon Eclipse 80i microscope.

### Investigation of Intestinal Microbiota by 16S RRNA Gene Sequencing

Roosters' feces were snap-frozen using liquid nitrogen, followed by storage at −80°C. DNA was extracted using the FastDNA™ Spin Kit for Feces (MP Biomedicals, Santa Ana, CA) following their manual. The purity and quality of the genomic DNA were checked in 0.8% agarose gels. The V3-V4 hypervariable regions of the 16S rRNA gene were amplified with the primers 338F (ACTCCTACGGGAGGCAGCAG) and 806R (GGACTACHVGGGTWTCTAAT) then sequenced using an Illumina NovaSeq 6000 System. The raw data were screened and sequences shorter than 200 bp, those with a low-quality score (≤ 20), those containing ambiguous bases, or those that did not exactly match to primer sequences and barcode tags were removed from consideration. Qualified sequences were separated using the sample-specific barcode sequences and trimmed with the Illumina Sequencing Analysis Pipeline Version 2.6. Afterward, the dataset was analyzed using QIIME. The sequences were clustered into operational taxonomic units (OTUs) at a similarity level of 97%. Taxonomic assignments of operational taxonomic units (OTUs) representative sequences were performed with a confidence threshold of 0.8 by a Naïve Bayes classifier trained on the GreenGenes database (version 13.8).

### Statistical Analysis

All statistical analyses were conducted using R (version 3.5.1) and GraphPad Prism (version 8.0), with all results reported as mean ± standard deviation (SD) or box-and-whisker plots. A one-way ANOVA test of multiple comparisons followed by Dunnett's *post-hoc* test was used to evaluate statistical significance between groups. Differences in the relative abundances of OTUs were assessed using Tukey's honest significant difference (HSD) test in R version 3.4.0. The level of statistical significance for our analyses is *p* < 0.05.

## Results

### GPS Improves SIgA Production in the Intestine

**Figure 1:**
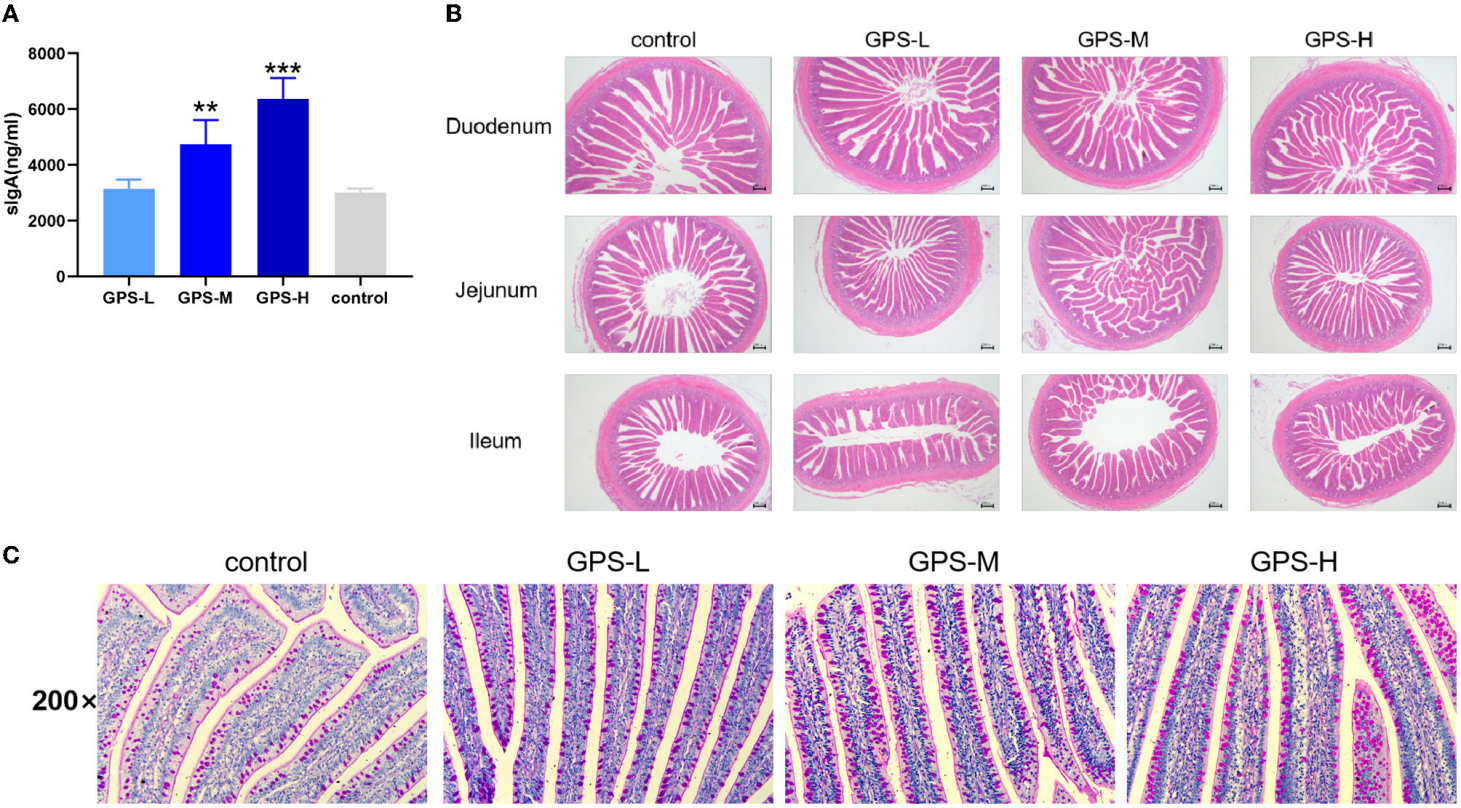
**(A)** Effects of 14-day oral administration of GPS on intestinal secretion of sIgA in laying hens. **(B)** Hematoxylin-Eosin (HE) staining was used to observe the effect of GPS on the histology of the small intestine of laying hens after 14 days of oral administration. **(C)** Glycogen was labeled using PAS staining to show goblet cells (darker granules) in the gut, and the effect of 14-day GPS oral administration on the number of intestinal goblet cells in laying hens. *P < 0.05, **P < 0.01, and ***P < 0.001, all relative to the saline (control) group.

To assess the effects of GPS on roosters' intestinal lining, the intestinal juice of roosters was collected to determine if GPS could increase local sIgA concentration. As shown in Figure 1A, sIgA was significantly increased in both the GPS-M and GPS-H treatment groups, relative to the control group. This suggests that GPS were able to boost production of sIgA in roosters' intestine in a dose-dependent manner.

### GPS Promotes an Increased Number of Small Intestinal Villi

The physical barrier of the intestine is composed of the lamina propria, the intestinal villi, the intestinal epithelium, and various tightly attached proteins in the intestinal cells. As shown in Figure 1B, the intestinal lamina propria was structurally intact and the small intestinal epithelium was healthy across all GPS treatment groups. There was also a significant and dose-dependent increase in the number of villi in the duodenum, jejunum, and ileum of the small intestine in the GPS groups.

### GPS Boosts Goblet Cell Secretion in the Intestine

The secretion activity of goblet cells in the intestine significantly affects the intestinal barrier and immunity (20). Thus, we further examined whether GPS could regulate goblet cell secretion in the intestine. As shown in Figure 1C, the secretion activity of goblet cells was significantly improved after treatment with GPS, compared to the saline group. This finding suggests that GPS are able to facilitate increased secretion from intestinal goblet cells, which promotes over-all intestinal health and a robust barrier against ingested pathogens.

### GPS Facilitates Production of Short-Chain Fatty Acids (SCFAs) in Intestines

**Figure 2:**
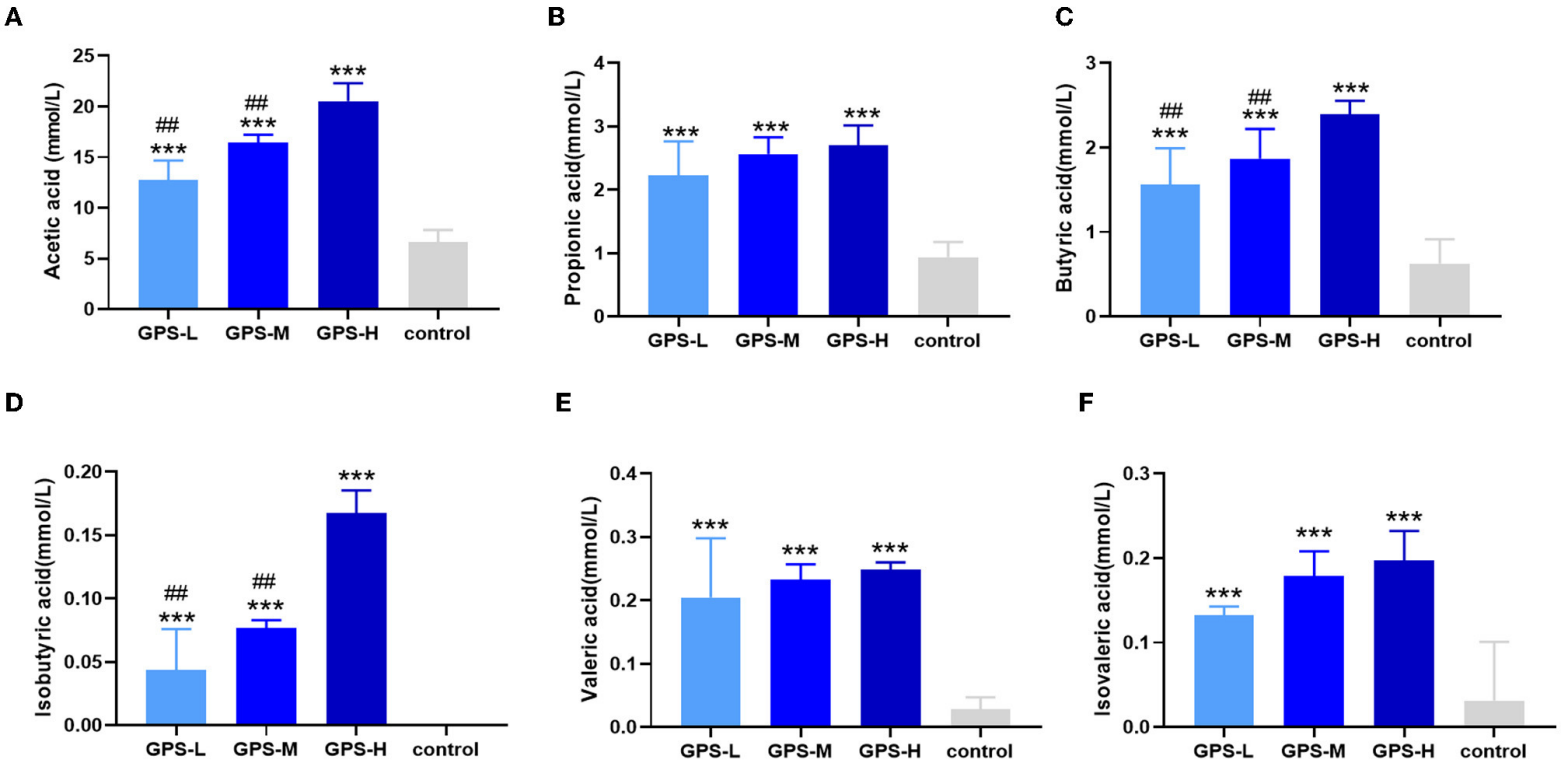
Effects of 14-day oral administration of GPS on SCFAs production in chicken intestines. The use of gas chromatography (GC) determined that after GPS treatment, significantly increased intestinal levels of **(A)** acetic acid, **(B)** propionic acid, **(C)** butyric acid, **(D)** isobutyric acid, **(E)** valeric acid, and **(F)** isovaleric acid content. *P < 0.05, **P < 0.01, and ***P < 0.001, all relative to the saline (control) group.

Short-chain fatty acids (SCFAs) including acetic acid, propionic acid, isobutyric acid, butyric acid, isovaleric acid, and valeric acid help regulate intestinal homeostasis and metabolism, which are key to intestinal health (21). Therefore, the content of these SCFAs in roosters' intestines was also examined. As shown in Figures 2A–F, the quantity of these SCFAs in the intestines of chickens from GPS-L, GPS-M, and GPS-H groups was significantly elevated compared with the control group, which suggests that GPS could promote the secretion of SCFAs. The high dose of GPS (GPS-H) elicited the strongest promotion effect compared to two lower GPS doses. Among them, the content of acetic acid, isobutyric acid, and butyric acid in the high-dose GPS group was significantly different from that in the low-dose group, indicating that this effect is dose- dependent.

### GPS Promotes Expression of Cytokines in the Intestine

**Figure 3:**
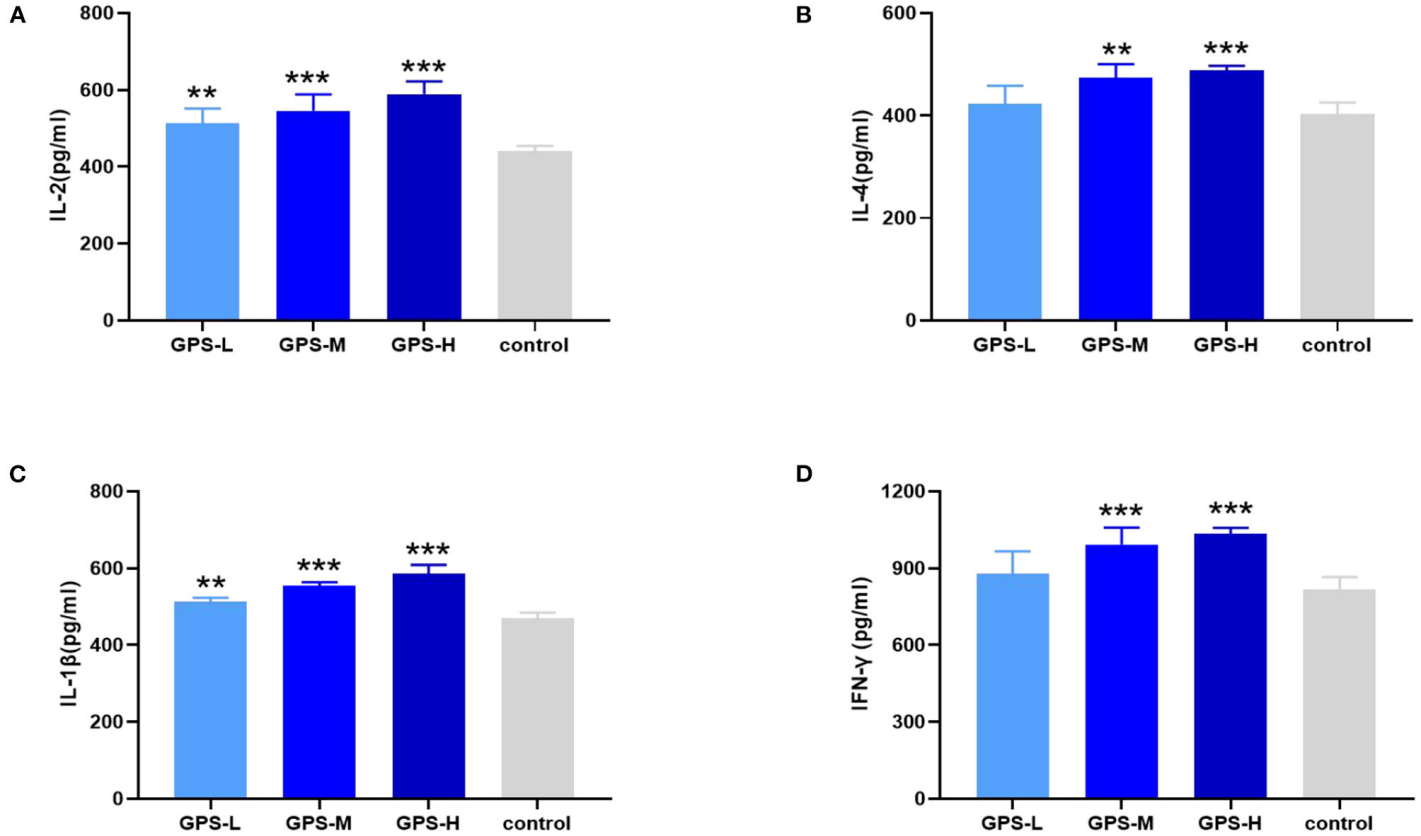
After the laying hens were orally administered GPS for 14 days, the changes of cytokines in the serum of laying hens were determined by ELISA.GPS enhances the expression of **(A)** IL-2, **(B)** IL-4, **(C)** IL-1β, and **(D)** IFN-γ. *P < 0.05, **P < 0.01, and ***P < 0.001, all relative to the saline (control) group.

Maintaining intestinal health and homeostasis is inseparable from the regulation of cytokines (22), which influence immune cells, tissue repair, and infection responses (23). As shown in Figures 3A,B, after treatment with GPS, levels of IL-2 and IL-4 were significantly elevated in the intestine compared to control group chickens treated with isotonic saline. After treatment with GPS, the production of IL-1β and IFN-γ was promoted in the intestine (see Figures 3C,D). These results suggest that GPS could enhance intestinal health through facilitation of IL-2, IL-4, IL-1β, and IFN-γ secretion.

### GPS Improves Expression of CD4+ and CD8+ T Cells in the Intestine

**Figure 4:**
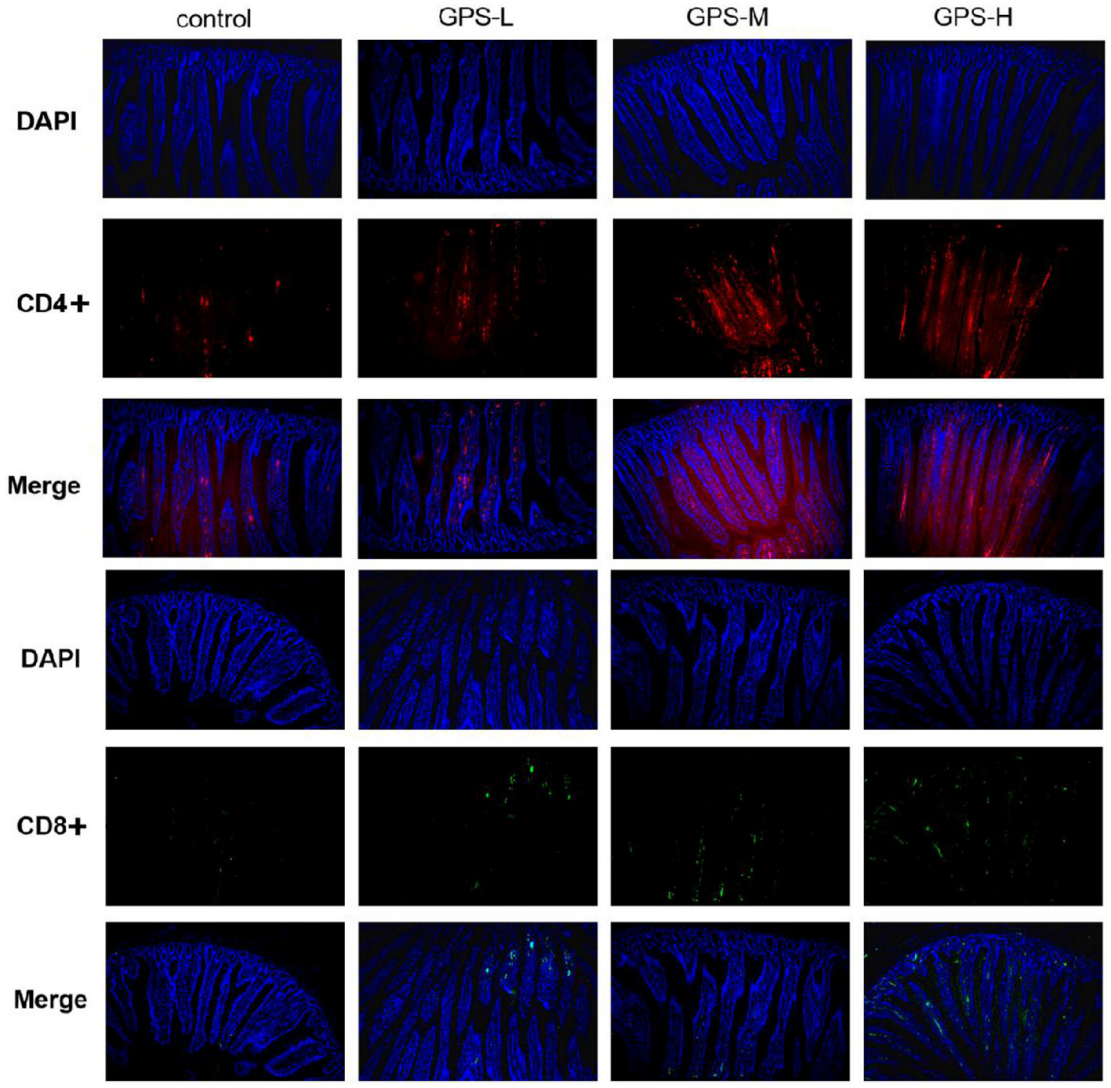
The proportion of CD4+ and CD8+ T cells in the gut of laying hens after 14 days of oral GPS was analyzed using immunofluorescence. The nuclei were stained with DAPI (blue), the cell surface differentiation antigen CD4+ was stained with CY3 (red), and the cell surface differentiation antigen CD8+ was stained with FITC (green), which is convenient for visual inspection of the fluorescence intensity.

Having confirmed the promotion effect of GPS on intestinal cytokine expression, we further explored whether GPS would affect activation of CD4+ and CD8+ T cells, which indirectly or directly prevent pathogen invasion and maintain homeostasis in the intestinal immune response (24). The immunofluorescence results in Figure 4 show that different doses of GPS could improve expressions of CD4+ and CD8+ to different extents, compared with saline treatments. Noticeably, the levels of both CD4+ and CD8+ were highest in the GPS-H group, suggesting both that GPS are able to promote the expression of CD4+ and CD8+ in the intestine, and that the effect is dose-dependent.

### GPS Enhances Beneficial Intestinal Microbiota Composition

**Figure 5:**
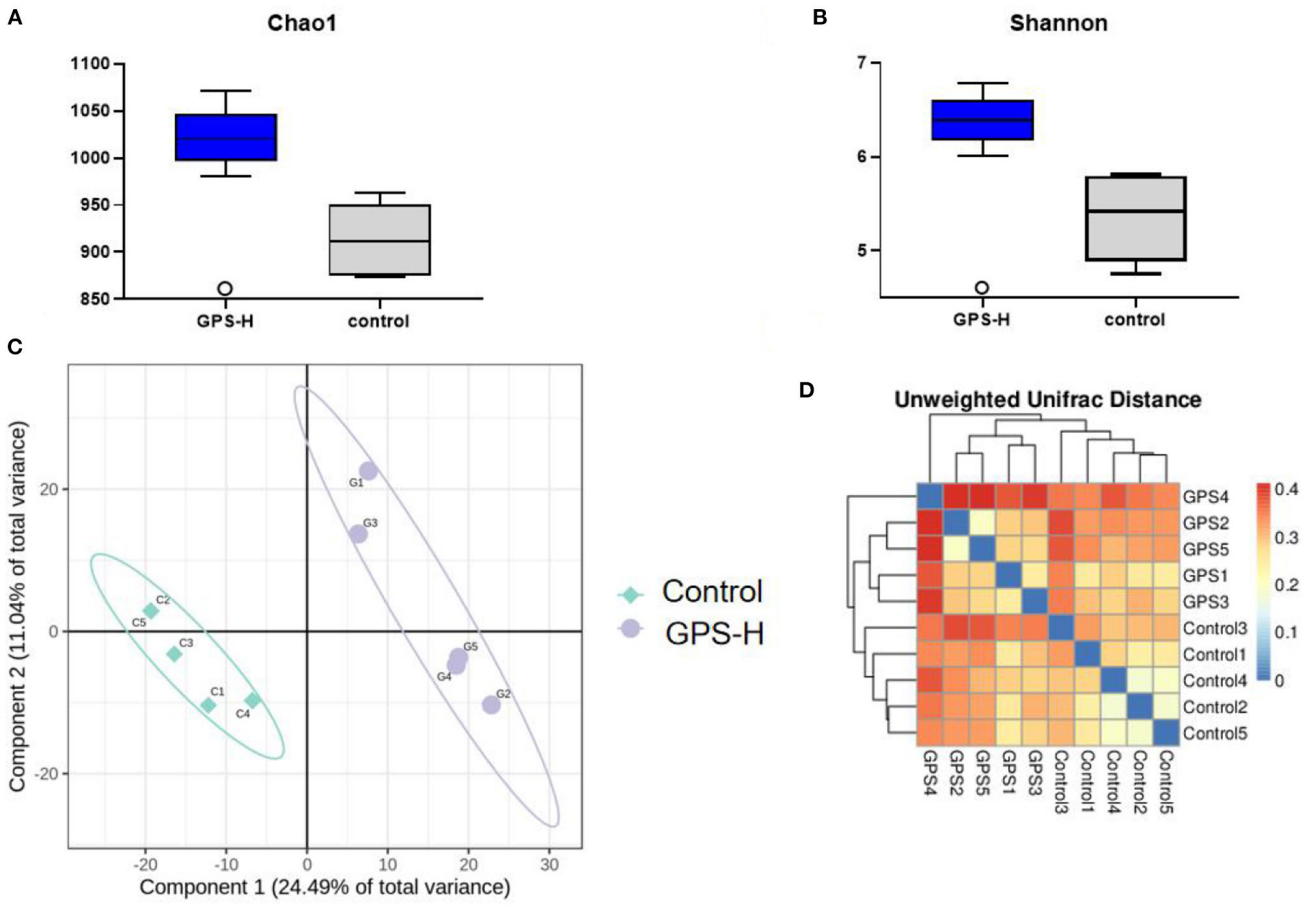
Changes in gut microbes in GPS-H-treated and untreated layers. **(A)** Chao1 and **(B)** Shannon indices represent microbial richness and evenness, respectively, represents the change in the alpha diversity of gut microbes between the two groups of samples. And **(C)** principal component analysis (PCA) and **(D)** heat map relationship (the horizontal and vertical axes are the names of the samples, and the color depth represents the similarity between samples.) The analysis represents the beta diversity between each sample variety.

The influence of GPS on the intestinal barrier and local immune response may be attributed to changes in intestinal microbiota. Therefore, the experiment further investigated intestinal microbiota for changes in evenness (uniformity) and richness (the number of species observed in each sample). As shown in Figures 5A,B, Shannon and Chao1 indices, which represent the evenness and total richness of intestinal microbiota, respectively, were both significantly increased in the GPS-H group relative to the control, suggesting that GPS-H could improve the evenness and richness of intestinal microbiota. Additionally, a principal component analysis (PCA) revealed that after treatment with GPS, the intestinal microbiota composition is fundamentally altered from that of the control group, indicating that GPS could dramatically change the intestinal microbial composition (Figures 5C,D). These changes in alpha diversity (Figures 5A,B) and beta diversity (Figures 5C,D) can be attributed to GPS treatment rather a chance variation in this specific sample collection.

**Figure 6:**
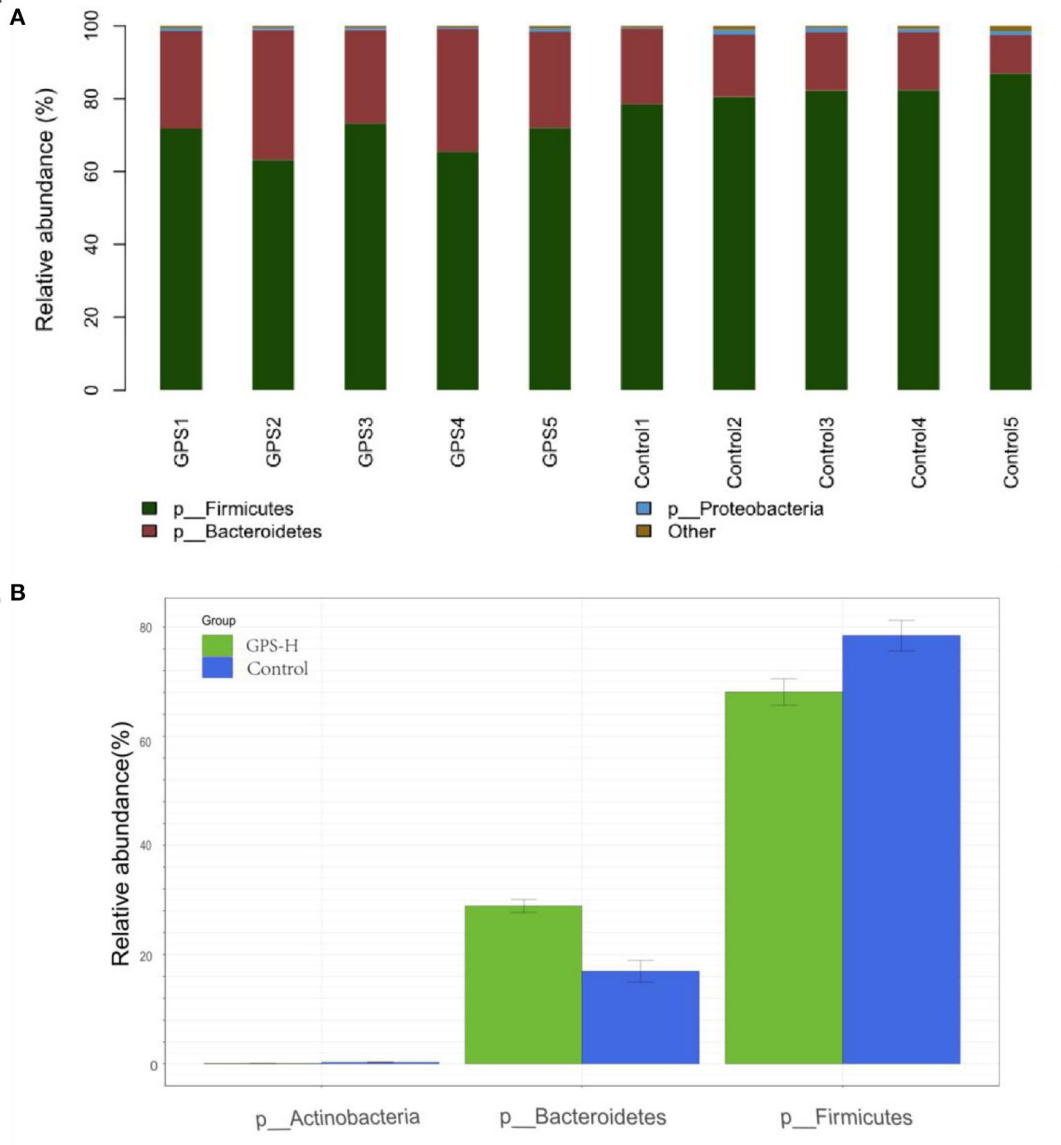
Analysis of significant differences between two groups was analyzed using Metastats analysis, which compares multiple samples under two conditions to find the types of microorganisms that are significantly different in the two groups. Analysis of intestinal microbiota composition by phylum. **(A)** The specific intestinal microbiota composition in individuals in GPS-H and control groups. **(B)** The relative abundance of intestinal microbes between GPS-H and control groups.

**Figure 7:**
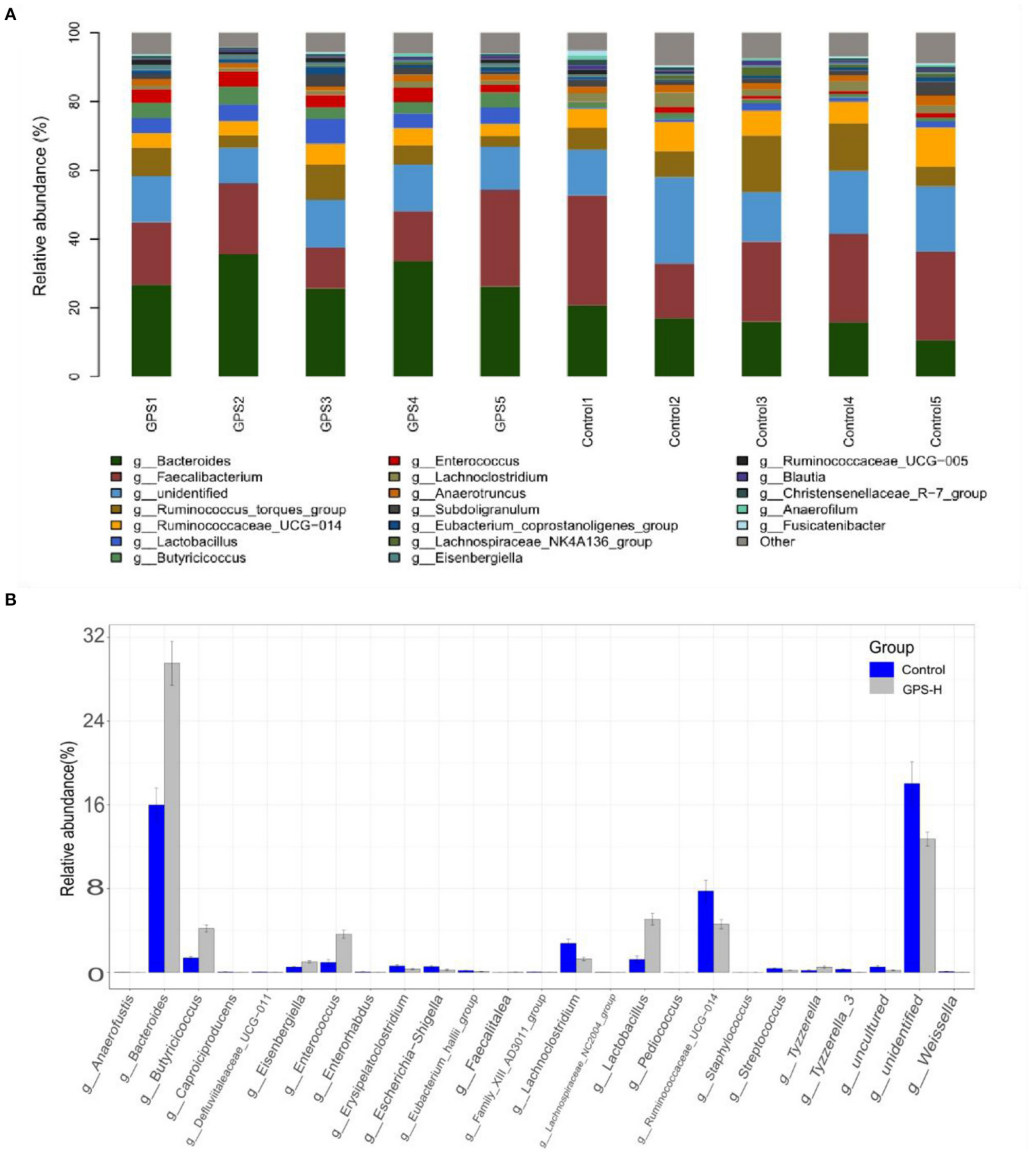
Analysis of significant differences between two groups was analyzed using Metastats analysis, which compares multiple samples under two conditions to find the types of microorganisms that are significantly different in the two groups.The specific composition of intestinal microbiota by genus. **(A)** The intestinal microbiota structure of each individual in GPS-H and control groups and **(B)** the average relative abundances of those intestinal microbes.

**Figure 8:**
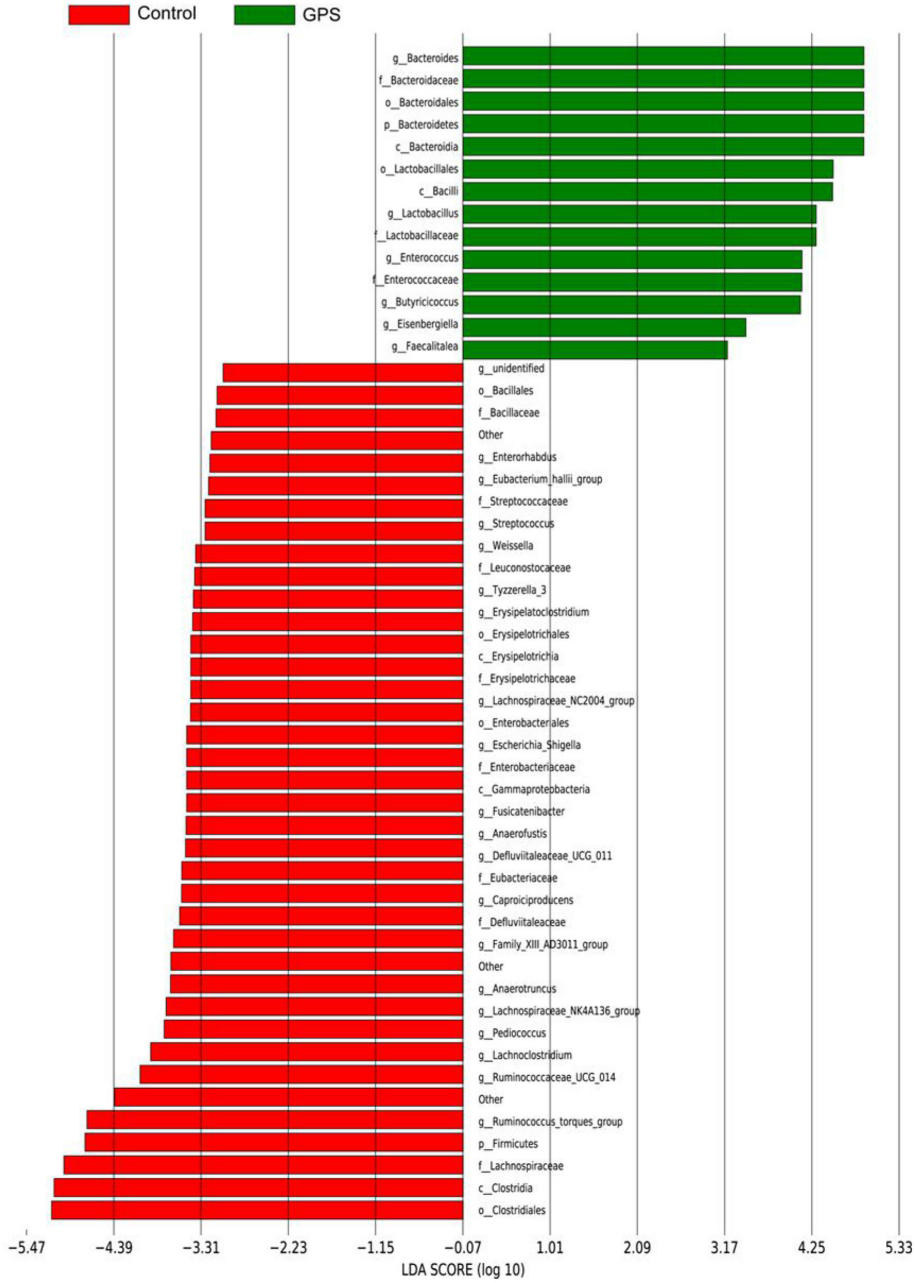
The LDA Effect Size was used to analyze the comparison between the GPS-H group and the control group, and an intra-subgroup comparison analysis between the two groups was also performed to find substances with significant differences in abundance between the groups.

The experiment further analyzed which specific intestinal microbes had changed to induce these alterations in alpha and beta diversity. From the phylum analysis shown in Figures 6A,B, it was observed that the proportion of Firmicutes were significantly decreased in the GPS-H group compared to the control. Meanwhile, relative to the control group, the proportion of Bacteroidetes was substantially increased in the GPS-H group; thus, the ratio of Firmicutes to Bacteroidetes was significantly reduced in the GPS-H group compared to the control. Notably, compared with roosters treated with saline alone, the proportion of Actinobacteria was increased in the intestines of roosters treated with GPS-H (Figure 6B), which suggests that GPS-H could not only reduce the ratio of Firmicutes to Bacteroidetes, but also regulate the proportion of specific microbes such as Actinobacteria. Further intestinal microbiota composition on a genus level is displayed in Figure 7. Bacteroides are the dominant flora in the intestines of roosters in the GPS-H group, and many microbes believed to be beneficial to the host were significantly increased in the GPS-H group compared to the control group (Figure 7A). These include Bacteroides, Butyricicoccus, Eisenbergiella, Enterococcus, Ruminococcaceae, and Lactobacillus. In addition, some pathogenic bacteria like Erysipelatoclostridium, Lachnoclostridium, and Escherichia–Shigella were significantly decreased in the GPS-H group compared to the control group (Figure 7B), suggesting that GPS are able to regulate the proportions of some harmful bacteria, which may contribute to the positive effect of GPS on roosters' intestinal health. Furthermore, this study defined which bacteria are primarily influenced by GPS using the method of linear discriminant analysis effect size (LEfSe.) As shown in Figure 8, the quantities of Bacteroidales, Bacteroidia, Lactobacillales, bacilli, Enterococcus, Butyricicoccus, and Eisenbergiella in the GPS-H group were significantly elevated relative to the control group, and the amounts of Clostridiales, Lachnospiraceae, Ruminococcaceae, Anaerotruncus, and Caproiciproducens were significantly decreased within the GPS-H group vs. the control, which accounts for the differences observed in Figure 5. These results taken together suggest that GPS-H could alter specific intestinal microbiota composition, and this change could help shape a better gut barrier and immune system.

### Administration of GPS Shows No Evidence of Toxicity

**Figure 9:**
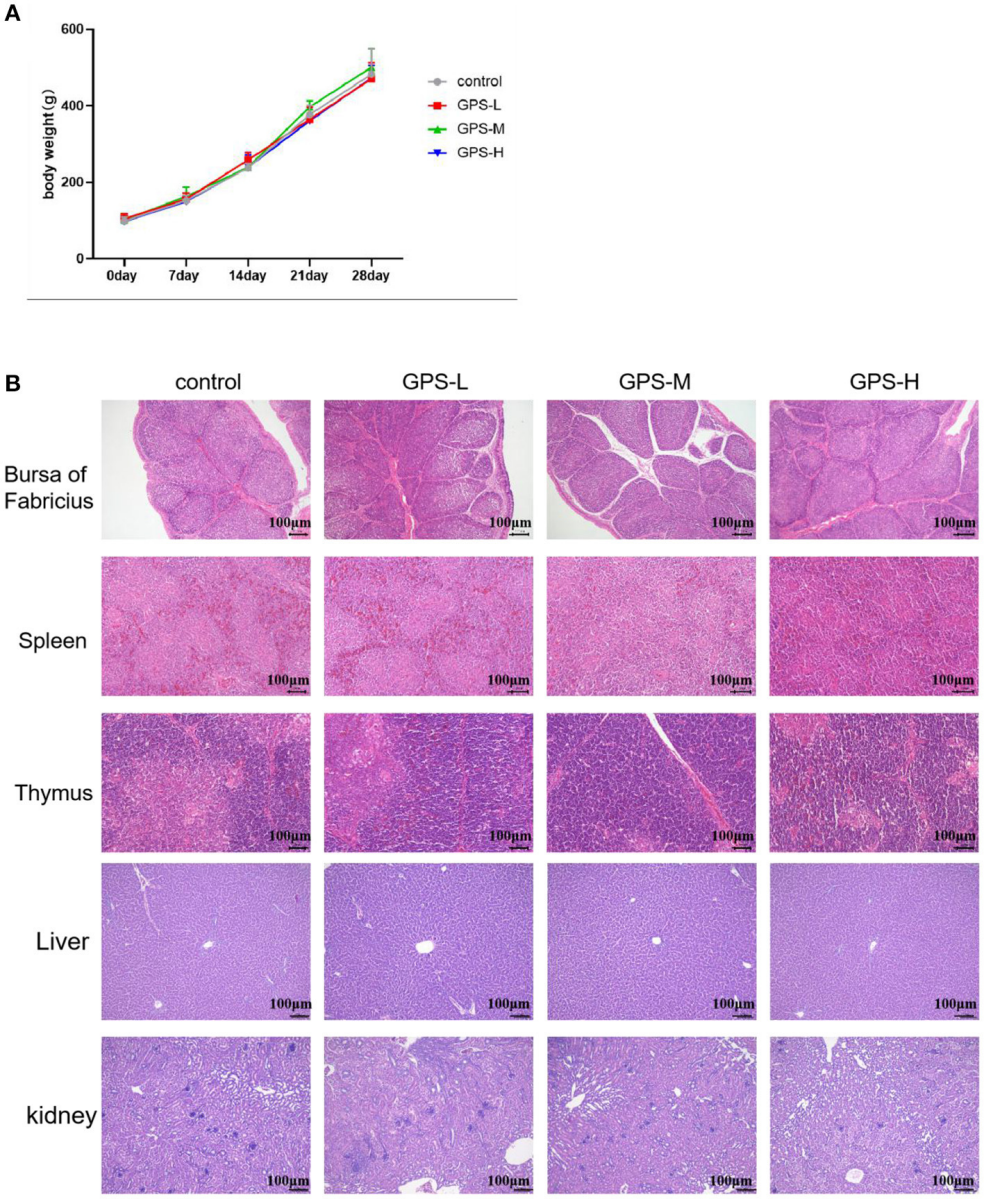
Examination of potential toxicity of GPS on roosters. **(A)** The influence of GPS on body weight. **(B)** The influence of GPS on immune organs (bursa of Fabricius, spleen, thymus, liver, and kidneys) after 14 days (100 × magnification, 100 μm scale bar).

Commercial feed additives are rightly scrutinized for safety as well as efficacy, thus, this study also examined the toxicity of GPS on roosters. For this purpose, body weight was monitored and the bursa of Fabricius, spleen, thymus, liver, and kidneys were examined to determine the potential toxicity of GPS. As shown in Figure 9B, there are no obvious pathological changes in the bursa of Fabricius, spleen, thymus, liver, or kidneys between the control and GPS-L, GPS-M, or GPS-H groups. Body weights all increased similarly; differences between the control and the three GPS groups (GPS-L, GPS-M, and GPS-H) are negligible, as illustrated in Figure 9A. These results indicate that GPS poses no toxicity as a feed additive for roosters.

## Discussion

Maintaining intestinal health and homeostasis is a complex balancing act influenced by many physiological factors in the interaction between the immune system, intestinal barrier, and intestinal microbiota (25). There are enormous quantities of bacteria living in animals' intestines, and differences in the composition of intestinal microbiota may dramatically alter the host's physiological and biochemical reactions, potentially destabilizing host homeostasis and general health (26). An animal's immune system recognizes and responds to the intestinal microbiota, which results in a moderate promotion of innate and adaptive immune pathways that reinforce the intestinal barrier and respond to invasive pathogens while maintaining tolerance to beneficial microbiota and antigens in food (27–29). These immune responses in the intestine help shape the intestinal microbiota composition by changing the nutrient substrates through mucus production or epithelial fucosylation (30). Thus, promoting the immune system, reinforcing the intestinal barrier, and improving intestinal microbiota are keys to enhancing animals' intestinal health. For the poultry farming industry, finding a potent feed additive to enhance animals' intestinal health is a useful strategy which can increase profits while lessening the risk of antibiotic resistant microbes threatening human health.

SIgA possesses many protective functions in the intestine, including as a defender to neutralize bacterial toxins in the gut lumen and disable viruses during transcytosis through the epithelial barrier (31). SIgA also inhibits abnormal epithelial cell translocation and excessive inflammatory responses induced by Shigella lipopolysaccharide (LPS) (32). In addition, sIgA can bind luminal bacteria into Peyer's patches (PPs) (33), which trains the immune system to tolerate beneficial bacteria and eliminate harmful bacteria (34). The present study verifies that GPS possess the ability to increase the sIgA content in the intestine, suggesting that GPS could promote roosters' intestinal health and beneficially alter intestinal microbiota. Goblet cells secrete mucus to protect the intestine and promote the expression of antimicrobial peptides that maintain intestinal homeostasis (20, 35). After treatment with GPS, secretion activity of goblet cells was significantly enhanced. This collective evidence leads to the conclusion that GPS are able to reinforce intestinal barrier health by increasing the content of sIgA and promoting the secretion activity of goblet cells.

Cytokines participate in the activation and derivation of immune cells, and thus are regarded as central to the immune system (36). Different cytokines exhibit diverse functions. They regulate the secretion of physiologically active substances and help maintain homeostasis. IL-2 and IL-4 are cytokines involved in appropriate immune responses and help avoid uncontrolled inflammation while promoting tissue repair (37). IL-1β and IFN-γ cytokines are able to protect the intestine from infection by triggering an immune response and preventing the spread of pathogens, respectively (38, 39). IL-2, a cytokine that can activate Tregs (T) cells and reinforce the inhibitory effect of Tregs on Teff, is also able to prevent the occurrence of spontaneous inflammation, and therefore plays a significant role in intestinal health (40). Our results show that GPS can promote the expression of IL-2, suggesting that GPS may regulate intestinal inflammation. IL-4, a typical Th2-type cytokine, has a potent anti-inflammatory effect (41). IFN-γ, a Th1-type cytokine, also exerts a protective effect on the intestine (42). The ratio of IL-4 to IFN-γ reflects the balance between Th2 and Th1 immune responses. The results show that GPS can increase the production of both IL-4 and IFN-γ, maintaining homeostasis in the intestine while boosting the overall immune response and promoting intestinal health. Having confirmed the effect of GPS on cytokine expression, we further explored whether immune cells in the intestine would be changed, and the proportion of CD4+ and CD8+ T cells were selected for this purpose. CD4+ and CD8+ T cells represent the Th1-type and Th2-type immune responses, respectively (43). The results showed that the numbers of both CD4+ and CD8+ T cells were increased after treatment with GPS, which is in agreement with the promotion effect on cytokine expression. Taken together, these results indicate that GPS can promote intestinal health by regulating the expression of cytokines and activation of CD4+ and CD8+ T cells while maintaining the homeostasis of Th1-type and Th2-type immune responses.

Given the interactions between sIgA, the immune system, and intestinal microbiota, our experiment further examined the effect of GPS on intestinal microbiota. It has been reported that sIgA can coat specific bacteria in the intestine, selectively enriching some members of the microbiota, as microbes vary in their propensity to acquire an sIgA coat when needed (44). Based on the increase of sIgA observed in our roosters treated with high-dose GPS (GPS-H), we further investigated the changes in these rooster's intestinal microbiotas. Many studies have concluded that the evenness and richness of intestinal microbiota are closely related to intestinal homeostasis, and the greater these measures, the more robust the immune response to pathogens (45). The results showed that GPS-H could increase both evenness and richness in the intestinal microbiota, suggesting that GPS-H could improve the host's ability to maintain intestinal homeostasis and improve immune responses. Also of note, the results show that GPS-H altered the composition of the intestinal microbiota, significantly improving the proportion of Bacteroidetes in the intestine, which could be attributed to the increase in sIgA allowing more Bacteroidetes to outcompete other microbes (46). Bacteroidetes break down complex polysaccharides like GPS into components like SCFAs and ferulic acid that they can metabolize, which would explain the jump in SCFAs in the intestine after GPS treatment (47) (Figures 2A–F). From the genus analysis, we found six bacteria significantly increased in the GPS-H group: Bacteroides, Butyricicoccus, Eisenbergiella, Enterococcus, Ruminococcaceae, and Lactobacillus. Butyricicoccus has been reported to promote the accumulation of butyric acid in the intestine (48). Meanwhile, there is evidence that chickens with a higher proportion of Eisenbergiella in their intestines possess a better metabolism and more efficiently use amino acids, nucleotides, and short-chain fatty acids (49). Enterococcus is a beneficial bacterium which plays a role in decomposing food and promoting the absorption of nutrients (50). Ruminococcaceae is the main microorganism that converts primary bile acids into secondary bile acids, which are severely lacking in patients with ulcerative colitis. Supplementation of Ruminococcaceae reduced inflammation and symptoms of colitis in mice (51). Lactobacillus is beneficial in treating irritable bowel syndrome (IBS) and reduces inflammation (51). Conversely, Erysipelatoclostridium, Lachnoclostridium, and Escherichia–Shigella are typical pathogenic bacteria in the intestine, and were seen in lower proportions after GPS-H treatment, compared to the control group. This suggests that GPS-H can effectively reduce the proportion of harmful bacteria in the intestine. Over-all, GPS-H treatment is able to regulate roosters' intestinal microbial composition in a manner beneficial for the host. Furthermore, roosters treated with GPS-H exhibited no signs of drug toxicity, which makes it possible for GPS to be used as a novel feed additive.

## Conclusions

GPS can improve intestinal health in three ways: improving the function of the intestinal barrier by increasing levels of sIgA and promoting goblet cell secretion, boosting the activation of the immune system by increasing the expression of cytokines and elevating levels of CD4+ and CD8+ T cells in the intestine while maintaining the homeostasis of Th1 to Th2 responses, and beneficially regulating the intestinal microbiota composition. These findings, combined with no evidence of toxicity, lead us to conclude that GPS shows promise as a novel feed additive and warrants further investigation as a substitute to prophylactic antibiotics in commercial poultry.

## Data Availability Statement

The original contributions presented in the study are included in the article/Supplementary Material, further inquiries can be directed to the corresponding author.

## Ethics Statement

The animal study was reviewed and approved by Nanjing Agricultural University No. PZ2020101.

## Author Contributions

YiW: conceptualization, funding acquisition, project administration, and supervision. YuW: data curation, writing—original draft preparation, and visualization. CW and YueW: data curation. YC: writing—original draft preparation and methodology. TZ: methodology and editing. CD: methodology. NL: data curation and visualization. KD: reviewing and editing. AN and YH: writing—reviewing and editing. HZ, XW, and HL: data validation. PH: editing. CL: writing—reviewing. All authors contributed to the article and approved the submitted version.

## Funding

This research was financially supported by the National Natural Science Foundation of China (NSFC, Grant No. 31872514 and 32172900), the Open Project Program of Bei-jing Key Laboratory of Traditional Chinese Veterinary Medicine at Beijing University of Agriculture (No. kf-tcvm202101), Yunnan Provincial Science and Technology Department-Applied Basic Research Joint Special Funds of Yunnan University of Chinese Medicine [2018FF001 (-020), 2019FF002(-012)], and a project funded by the Priority Academic Program Development of Jiangsu Higher Education Institutions (PAPD). We appreciate the assistances from our distinguished colleagues in the Institute of Traditional Chinese Veterinary Medicine of Nanjing Agricultural University.

## Conflict of Interest

KD was employed by China Tobacco Henan Industrial Co. Ltd. The remaining authors declare that the research was conducted in the absence of any commercial or financial relationships that could be construed as a potential conflict of interest.

## Publisher's Note

All claims expressed in this article are solely those of the authors and do not necessarily represent those of their affiliated organizations, or those of the publisher, the editors and the reviewers. Any product that may be evaluated in this article, or claim that may be made by its manufacturer, is not guaranteed or endorsed by the publisher.
